# High-frequency variants in PKA signaling-related genes within a large pediatric cohort with obesity or metabolic abnormalities

**DOI:** 10.3389/fendo.2023.1272939

**Published:** 2023-11-13

**Authors:** Michelle Bloyd, Ninet Sinaii, Fabio Rueda Faucz, James Iben, Steven L. Coon, Sonia Caprio, Nicola Santoro, Constantine A. Stratakis, Edra London

**Affiliations:** ^1^ Section on Endocrinology and Genetics, Program on Developmental Endocrinology and Genetics, Eunice Kennedy Shriver National Institute of Child Health and Human Development (NICHD), Bethesda, MD, United States; ^2^ Biostatistics and Clinical Epidemiology Service, National Institutes of Health (NIH) Clinical Center, Bethesda, MD, United States; ^3^ Molecular Genomics Core, National Institute of Child Health and Human Development (NICHD), Bethesda, MD, United States; ^4^ Section on Pediatric Endocrinology and Diabetes, Yale University, New Haven, CT, United States; ^5^ Department of Medicine and Health Sciences, “V. Tiberio” University of Molise, Campobasso, Italy; ^6^ Human Genetics and Precision Medicine, Institute for Molecular Biology and Biotechnology (IMBB), Foundation for Research & Technology Hellas (FORTH), Heraklion, ELPEN Research Institute, Athens, Greece

**Keywords:** obesity, protein kinase A, pediatric, liver disease, targeted exome sequencing, GPCRs, T2DM, genetics

## Abstract

**Introduction:**

Pediatric obesity has steadily increased in recent decades. Large-scale genome-wide association studies (GWAS) conducted primarily in Eurocentric adult populations have identified approximately 100 loci that predispose to obesity and type II diabetes. GWAS in children and individuals of non-European descent, both disproportionately affected by obesity, are fewer. Rare syndromic and monogenic obesities account for only a small portion of childhood obesity, so understanding the role of other genetic variants and their combinations in heritable obesities is key to developing targeted and personalized therapies. Tight and responsive regulation of the cAMP-dependent protein kinase (PKA) signaling pathway is crucial to maintaining healthy energy metabolism, and mutations in PKA-linked genes represent the most common cause of monogenic obesity.

**Methods:**

For this study, we performed targeted exome sequencing of 53 PKA signaling-related genes to identify variants in genomic DNA from a large, ethnically diverse cohort of obese or metabolically challenged youth.

**Results:**

We confirmed 49 high-frequency variants, including a novel variant in the PDE11A gene (c.152C>T). Several other variants were associated with metabolic characteristics within ethnic groups.

**Discussion:**

We conclude that a PKA pathway-specific variant search led to the identification of several new genetic associations with obesity in an ethnically diverse population.

## Introduction

Over the past decade, several genome-wide association studies (GWAS) in adults, but only a few in pediatric patient cohorts, have been conducted to uncover genetic loci associated with obesity and other increasingly prevalent pediatric metabolic diseases including non-alcoholic fatty liver disease (NAFLD) and type II diabetes (T2D). From these studies, approximately 100 identified genetic loci have been associated with body mass index (BMI) variability, the main correlating factor to obesity used in GWAS (1). However, investigation into the translatability of genetic risk loci in adults to pediatric patients has been spotty, with few genetic loci being associated with BMI variability in pediatric patients (1). While obesity is the most important risk factor for NAFLD, not all obese children develop NAFLD, demonstrating the importance of genetic factors in disease susceptibility. Additionally, most large-scale studies and genetic databanks are Eurocentric (2, 3), despite the 58% and 49% increased prevalence of obesity among Hispanic and non-Hispanic Black compared to non-Hispanic White children in the U.S (4). With the alarming, unabated increase in prevalence of childhood and adolescent obesity and other metabolic diseases, thorough investigation of all avenues leading to understanding the mechanistic underpinnings and genetic contributors is warranted.

The prevalence of T2D increased from 0.34 to 0.67 per 1,000 youths aged 10 to 19 years between 2001 and 2017, with the greatest increases observed in non-Hispanic Black and Hispanic youths (5). The prevalence of T2D is higher among those 10 to 19 years old compared to younger children, in part because of pubertal surges in growth hormone and insulin-like growth factor that promote insulin resistance (6). Youth-onset T2D is typically more aggressive, involves more frequent complications, and is less responsive to treatment than adult-onset T2D. As with NAFLD, obesity is the most important risk factor for T2D, yet not all obese children develop the disease, urging a better understanding of genetic differences in disease susceptibility (7). A number of common genetic loci that predispose to the development of T2D include genes involved in glucose production, insulin signaling or sensitivity, β-cell function, pancreatic development, and those that cause monogenic T2D (8). The use of polygenic risk scores (PRS) to predict T2D risk is an effective tool that gains utility as knowledge of risk variants expands. The cAMP-dependent protein kinase (PKA)/cAMP signaling pathway is an important target for T2D therapy development because of its central role in glucose homeostasis, including insulin and glucagon secretion, glycogen synthesis and breakdown, and gluconeogenesis (9).

NAFLD is the leading cause of chronic liver disease among children and adults and one of the main manifestations of metabolic syndrome (10, 11). NAFLD is a disorder with a wide spectrum, ranging from fat accumulation in >5% of hepatocytes to non-alcoholic steatohepatitis (NASH), steatofibrosis, and potentially cirrhosis, although the progression of disease is not well-documented in children (11). Genetic variation in the gene patatin-like phospholipase domain-containing protein 3 (*PNPLA3*) confers susceptibility to nonalcoholic fatty liver disease. The *PNPLA3* I148M variant, common among Hispanic populations, is associated with two-fold higher hepatic fat content than non-carriers while the S453I variant, common in African Americans (AAs), is associated with decreased hepatic fat (12). Several other well-known variants in the genes transmembrane 6 superfamily member 2 (*TM6SF2*) (variant: E167K), glucokinase regulator (*GCKR*), membrane bound O-acyltransferase domain containing 7 gene–transmembrane channel-like 4 (*MBOAT7-TMC4*) (variant: rs641738), and hydroxysteroid 17-beta dehydrogenase 13 (*HSD17B13*) (variant: rs72613567:TA), in addition to gut microbiota composition, contribute to NAFLD susceptibility in children (13). The etiology of NAFLD involves genetic and non-genetic factors, including oxidative stress; altered lipid metabolism, cytokines, and adipokines; and mitochondrial dysfunction that promotes inflammation, cell death, and fibrosis, that interact in parallel to increase NAFLD susceptibility (14). Dysregulated hepatic lipid metabolism, a key component of NAFLD, by default involves changes along the PKA/cAMP signaling pathway. Liver-specific PKA inhibition in mice altered the regulation of nearly 100 genes involved in hepatic lipid metabolism and reduced hepatic triglyceride accumulation significantly after chronic high-fat diet exposure (15). Other work showed that hepatic activation of PKA and, in turn, AMP-activated protein kinase (*AMPK*), through the PKA catalytic subunit α, stimulates lipolysis and protects against dysregulated hepatic lipid metabolism and apoptosis typically induced by free fatty acids or high-fat/high-sugar diet (16). Atherosclerotic cardiovascular disease (ASCVD) is the leading cause of adult morbidity and mortality in developed countries and begins in childhood with subclinical vascular changes (17). Dyslipidemia, diabetes, and obesity are primary risk factors for ASCVD and NAFLD, and the etiologies of both diseases involve combined genetic and lifestyle factors.

With multiple roles in glucose homeostasis and in lipid metabolism, storage, and trafficking, appropriate regulation of PKA/cAMP signaling is a common thread underlying the maintenance of healthy metabolic functions. Targeting PKA and cAMP signaling to develop obesity, T2D and dyslipidemia therapeutics is not a novel idea (18). Nearly 20% of the 361 drugs in clinical trials documented by the U.S Food and Drug Administration in 2017 were compounds targeting G-protein-coupled receptors (GPCRs) that act just upstream of the PKA/cAMP signaling pathway (19). PKA signaling is integral in regulating various aspects of energy homeostasis from adipocyte and liver biology to the central control of intake (20). Because of the intimate relationship between PKA and the maintenance of metabolic health, we sought to identify variants in PKA signaling-related genes (**Table 1**) that are involved in metabolic regulation among our predominantly obese pediatric cohort. The diversity of the cohort lends strength to the study and highlights the importance of ethnic background on the metabolic phenotype.

**Table 1:** Gene list for targeted exome sequencing including PKA subunit genes and other genes coding for proteins involved in the PKA signaling pathway (*n* = 53).

*ABCA1*	*CREB1*	*CYP27A1*	*GYS2*	*PDE1B*	*PDE11A*	*PRKAR2A*
*ADCY1*	*CREB3*	*CYP46A1*	*IL10RB*	*PDE2A*	*PON1*	*PRKAR2B*
*ADCY2*	*CREB3L1*	*DHCR24*	*IL6*	*PDE3B*	*PPARGC1B*	*PRKX*
*ADCY4*	*CREB3L2*	*DHCR7*	*MC2R*	*PDE4A*	*PRKACA*	*SLC2A2*
*ADCY5*	*CREB3L3*	*ERN1*	*MC3R*	*PDE4B*	*PRKACB*	*TM7SF2*
*AKAP7*	*CREB3L4*	*FGF21*	*MC4R*	*PDE7B*	*PRKACG*	
*APOE*	*CREM*	*G6PC*	*NR1H2*	*PDE8B*	*PRKAR1A*	
*ARPP19*	*CRH*	*GSK3B*	*NR1H3*	*PDE10A*	*PRKAR1B*	

Selection focused on genes known to regulate energy metabolism and lipid synthesis, trafficking, and storage.

## Methods

### Patient studies

The patients in this study were recruited from the Yale Obesity Clinic as part of the Pathogenesis of Youth Onset Diabetes (PYOD) cohort (NCT01967849). Participants were excluded if they had been previously treated for other endocrinopathies, were on any chronic medications known to alter glucose or insulin metabolism, or had been or were currently being treated with oral glucocorticoids, antirejection medication, or chemotherapy. Youth with a BMI between the 85th and 95th percentiles were defined overweight and youth with a BMI greater than the 95th percentile were defined obese. BMI percentiles were derived from age and sex national standards. Our study protocol was approved by the Human Investigations Committee of the Yale School of Medicine. Participants provided assent and parents provided written informed consent to participate in the study.

### Cohort demographics and clinical features

Genomic DNA from a cohort of 460 patients was sequenced and association analyses based on known gene functions were performed. Data for 86 clinical parameters were available for most patients including hepatic fat fraction (HFF); anthropometric data; blood pressure; fasting levels of glucose, insulin, and C-peptide and their levels during oral glucose tolerance test (OGTT); homeostatic model assessment (HOMA), whole-body insulin sensitivity index (WBISI), insulinogenic index (IGI), disposition index (DI), and glycosylated hemoglobin (HbA1c); expanded cholesterol, chylomicron, and triglyceride values including very-low-density lipoprotein (VLDL), high-density lipoprotein (HDL), and low-density lipoprotein (LDL) size; serum glutamic oxaloacetic transaminase (SGOT), serum glutamic pyruvate transaminase (SGPT), gamma glutamyl transferase (γ-GTT), alkaline phosphatase; and apolipoprotein B (ApoB).

### Clinical data collection

Magnetic resonance imaging (MRI) studies were performed on a GE or Siemens Sonata 1.5 Tesla system. Measurement of liver fat content was performed by MRI using the two-point Dixon (2PD) method as modified by Fishbein et al. (21). HFF% was calculated in duplicate from the mean pixel signal intensity data using the formula: [(S_in_−S_out_)/(2×S_in_)]×100. The imaging parameters included the following: matrix size = 128 × 256, flip angle = 30°, TR = 18 ms, TEs = 2.38/4.76 ms out-of-phase and in-phase, respectively, bandwidth = 420 Hz/pixel, six averages, slice thickness = 10 mm, one slice, 2.3 s/slice (for 2 points), scan time = 14 s in a single breath-hold.

A standard OGTT (1.75 g/kg body weight, up to 75 g) was performed. Blood samples were drawn at −15-, 0-, 30-, 60-, 90-, 120-, and 180-min time points for determination of glucose, insulin, and C-peptide. Insulin sensitivity was assessed by the WBISI (22). The WBISI was calculated as 10,000/square root of [(fasting glucose × fasting insulin) × (mean glucose × mean insulin during OGTT)] (2). The IGI, an index of early-phase insulin secretion, was calculated as: IGI = insulin (0–30 min) in microunits per milliliter divided by the glucose (0–30 min) in milligrams per deciliter. The DI, which provides a picture of the beta cell response in an insulin-resistant milieu, was calculated as the product of the IGI and the WBISI, based on the curvilinear relation of these OGTT-derived parameters previously described by our group in obese children and adolescents (22, 23).

Plasma glucose was determined using a glucose analyzer by the glucose oxidase method (Beckman Instruments, Brea, CA). Plasma insulin was measured by the Linco radioimmunoassay, lipid levels were determined with an auto-analyzer (model 747-200), and liver enzymes were measured using standard automated kinetic enzymatic assays.

Fasting plasma samples were obtained to determine lipoprotein particle concentrations and sizes. The analyses were conducted with a 400-MHz proton NMR analyzer at Liposcience (Raleigh, NC), and the methodology used has been previously described (24). Briefly, each lipoprotein subclass concentration was measured by the amplitudes of the characteristic lipid-methyl group NMR signals that they emit. The intensity of each signal was proportional to the quantity of the subclass, which was reported in particle concentration units (nmol/L for VLDL and LDL and μmol/L for HDL). VLDL, LDL, and HDL were separated into 10 subclass categories: large VLDL (including chylomicrons) (>60 nm), medium VLDL (35–60 nm), small VLDL (27–35 nm), intermediate-density lipoprotein (IDL) (23–27 nm), large LDL (21.2–23 nm), medium-small LDL (19.8–21.2 nm), very small LDL (18–19.8 nm), large HDL (8.8–13 nm), medium HDL (8.2–8.8 nm), and small HDL (7.3–8.2 nm). Average particle sizes were computed as the sum of the diameter of each subclass multiplied by its relative mass percentage as estimated from the amplitude of its methyl NMR signal.

### Classification of NAFLD, glucose tolerance status, and dyslipidemia

NAFLD was defined as having greater than or equal to 5% of hepatocytes containing macrovesicular fat (HFF% ≥ 5) as quantified by the MRI method previously described. Glucose tolerance status was considered normal, impaired, or T2D at the following fasting glucose levels: 60–100 mg/dL, 100–125 mg/dL, and greater than 125mg/dL. Dyslipidemia was characterized as having any of the following: total cholesterol of greater than or equal to 200 mg/dL; low density lipoprotein (LDL) cholesterol of greater than or equal to 130 mg/dL; triglycerides greater than or equal to 100 mg/dL (0–9 years old) and greater than or equal to 130 mg/dL (10–18 years old); or high-density lipoprotein (HDL) cholesterol less than 40 mg/dL.

### Targeted exome sequencing and data compilation

Targeted exome sequencing and its pursuant data compilation were completed as previously described (25). In short, custom amplicons for 53 PKA signaling-related genes (**Table 1**) were created with Illumina DesignStudio; TruSeq Custom Amplicon Library Preparation Kit was used to generate the cDNA libraries that were then sequenced with MiSeq (Illumina; v2 chemistry). Call sets were generated and annotated as previously reported (25).

### Data processing and *in silico* prediction

All data compiled from the targeted exome sequencing was systematically reviewed and categorized as described before (25). Briefly, only variants with a high allele frequency (defined as a frequency greater than or equal to 0.004) and a predicted high or moderate physiological impact were further investigated. The database Varsome.com was used to gather detailed information on variants of interest and Integrative Genomics Viewer (IGV_2.4.16, Broad Institute) was used to determine the legitimacy of variants. Variants of interest that passed this scrutiny were confirmed with Sanger sequencing.

### Statistical analyses

Data are described using frequency (percentages), and simple descriptive statistics of mean ± standard deviation (SD) or median (inter-quartile range, IQR). Data were assessed for distributional assumptions and analyzed accordingly. Mixed models were used for comparing demographic and outcome data by race/ethnicity reported in **Table 2**. In addition, they tested associations between the main effect of race/ethnicity and outcomes (e.g., WBSI, HDL, and VLDL) and adjusted for appropriate covariates (e.g., BMI and NAFLD) as necessary. Statistical evidence was based on effect sizes, variability, and *p*-values. Data were analyzed using SAS v9.4 (SAS Institute, Inc, Cary, NC).

**Table 2:** Patient demographics and clinical characteristics.

	Total^1^	Caucasian	African American	Hispanic	Other	*p*-value^2^
***n***		**175 (38.0%)**	**118 (26.7%)**	**138 (30.0%)**	**29 (6.3%)**	
Sex						<0.001
Female	261 (56.7%)	106 (60.6%)	75 (63.6%)	58 (42.0%)	22 (75.9%)	
Male	199 (43.3%)	69 (39.4%)	43 (36.4%)	80 (58.0%)	7 (24.1%)	

Age, median (IQR)	13.3 (11.1–16.0)	14.0 (10.9–16.1)	13.4 (11.6–16.2)	12.8 (10.7–14.9)	13.0 (11.4–15.1)	0.080

Obesity	354 (81.8%)	131 (79.4%)	89 (82.4%)	113 (84.3%)	21 (80.8%)	0.74
BMI Percentile	98.7 (96.8–99.4)	98.6 (95.8–99.4)	98.9 (97.1–99.5)	98.7 (97.2–99.3)	98.5 (95.3–99.0)	0.58
Body Fat	42.0 (34.7–47.4)	41.6 (34.6–46.8)	44.2 (35.9–48.8)	41.4 (33.4–46.5)	41.2 (31.7–46.4)	0.50
Waist Circumference	98.0 (87.5–110.0)	98.0 (88.0–111.0)	101.0 (87.0–113.0)	98.0 (89.0–106.0)	94.0 (80.0–101.0)	0.23
Visceral Fat	50.3 (31.8–71.5)	54.2 (35.6–75.8)	40.3 (24.1–59.2)	53.0 (37.2–75.4)	48.3 (35.2–71.5)	0.002

NAFLD	165 (36.0%)	60 (34.5%)	35 (29.7%)	59 (43.1%)	11 (39.3%)	0.15
HFF %	2.5 (0–9.6)	3.0 (0–8.8)	0 (0–2.8)	6.2 (0.8–17.8)	2.3 (0–8.8)	<0.001
Glucose Tolerance^3^						
Normal (NGT)	352 (76.5%)	139 (79.4%)	84 (71.8%)	105 (76.1%)	24 (82.8%)	0.59
Impaired (IPGT)	96 (20.9%)	31 (17.7%)	30 (25.6%)	31 (22.5%)	4 (13.8%)	
T2D	12 (2.6%)	5 (2.9%)	3 (2.6%)	2 (1.5%)	1 (3.5%)	
HOMA	6.2 (4.1–9.1)	6.2 (4.1–8.6)	6.3 (4.2–10.7)	6.1 (4.1–9.1)	6.2 (4.2–8.0)	0.92
DI	5.7 (3.7–8.7)	5.1 (3.3–7.7)	7.2 (4.2–11.3)	5.8 (3.6–8.9)	5.3 (3.7–7.8)	0.043

Dyslipidemia^4^	221 (50.3%)	83 (50.3%)	46 (40.4%)	82 (61.2%)	10 (38.5%)	0.006
Total Cholesterol	153 (134–174)	150 (132–169)	156 (138–176)	147 (128–173)	157.5 (137–183)	0.91
Triglyceride	85 (61–126)	91 (65–141.5)	64 (49–95)	94.5 (71–141)	99.5 (73–124)	<0.001

Data are bolded n (%), with each ethnic/racial group as the numerator and the total number of study patients as the denominator for calculating percentages.

Data were not approximately normally distributed among ethnic/racial groups and are presented as median (IQR).

IQR, interquartile (25th–75th percentile) range; BMI, body mass index; HFF %, hepatic fat fraction percent; NGT, normal glucose tolerance; IPGT, impaired glucose tolerance; T2D, type II diabetes.

^1^ n = 1 patient was missing ethnic/race information and was excluded from all analyses by race.

^2^ p-values are from comparisons among ethnic/racial groups.

^3^ n = 1 patient was missing data on glucose tolerance.

^4^ Dyslipidemia based on the following definition: total cholesterol of ≥200 mg/dL; LDL-C of ≥130 mg/dL; TG ≥ 100 mg/dL (0–9 years old), ≥130 mg/dL (10–18 years old); or HDL–C < 40 mg/dL.

## Results

### Cohort demographics and metabolic characteristics

In our cohort, 38% of patients (*n* = 175) were Caucasian, 26.7% (*n* = 118) were AA, 30% (*n* = 138) were Hispanic, and 6.3% (*n* = 29) were Asian or “other” (**Table 2**). Female and male patients represented 56.7% and 43.3% of the patient cohort, respectively (*p* < 0.001). Median patient age was 13.3 (IQR 11.1–16.0) years old. Within the cohort, 36.0% of patients had NAFLD, 20.9% had impaired glucose tolerance, 2.6% had T2D, and 50.3% had dyslipidemia. Most of the cohort was obese (81.8%) with a median BMI percentile of 98.7. Finally, 139 patients harbored at least one confirmed variant in the targeted PKA signaling-related genes (**Table 3**). Of these patients, 18% (*n* = 25) were Caucasian, 58% (*n* = 81) were AA, 21% (*n* = 29) were Hispanic, and 3% (*n* = 4) were Asian or “other”. Breakdown by sex revealed that 58% (*n* = 81) of variant-harboring patients were female and 42% (*n* = 58) of variant-harboring patients were male. While the ratio by sex among Hispanic variant-harboring patients was 38% male (*n* = 11) to 62% female (*n* = 18), ratios by sex among other ethnic groups followed the trend seen in the total variant-harboring patient population.

**Table 3:** Complete list of confirmed variants and their breakdown by ethnic group and sex, (n= female:male).

Gene	Variant Code	Ethnic and Sex Breakdown (n= female:male)	Gene	Variant Code	Ethnic and Sex Breakdown (n= female:male)	Gene	Variant Code	Ethnic and Sex Breakdown (n= female:male)
ABCA1	c.2602G>A	AA (3:1)	CREB3L1	c.599A>T	Caucasian (1:3)	MC4R	c.392_393delGC	AA (1:0)
	c.2089G>A	AA (3:1), Hispanic (1:1)		c.1333A>G	AA (1:0), Hispanic (0:1)	PDE1B	c.22C>T	Caucasian (1:0), Hispanic (0:1)
	c.1765G>A	Hispanic (2:1)		c.1547T>G	Hispanic (1:0)	PDE2A	c.608C>T	AA (2:2), other (1:0)
	c.634T>A	AA (5:0)	CREB3L4	c.142C>T	Caucasian (0:1)	PDE3B	c.1242G>C	AA (1:1), Hispanic (0:1)
	c.2311G>C	AA (1:0)	CREM	c.2T>C	Hispanic (1:0)	PDE4A	c.1588C>T	AA (3:0), Hispanic (0:1)
	c.5449C>T	Hispanic (0:1)	CYP27A1	c.524C>T	AA (1:0), Hispanic (0:1)		c.163C>G	AA (4:4)
ADCY4	c.-26-1G>T	AA (1:3)	CYP46A1	c.1210T>C	AA (0:1)	PDE7B	c.1347C>G	AA (3:2)
	c.1975C>T	Hispanic (0:1)		c.1444G>T	Hispanic (1:0)	PDE10A	c.1658G>A	Caucasian (0:1)
ADCY5	c.29C>T	AA (1:3), Hispanic (1:1)	DHCR7	c.199G>A	Caucasian (3:0), AA (1:0)	PDE11A	c.152C>T	Caucasian (4:4), AA (1:0), Hispanic (2:0)
AKAP7	c.76G>A	AA (6:4), Hispanic (0:1)		c.70G>T	AA (2:1), Hispanic (0:1)	PPARGC1B	c.2557C>T	Caucasian (2:2)
	c.439G>T	AA (7:2), Hispanic (1:4)	ERN1	c.1142A>G	AA (2:2), Hispanic (0:1), other (0:1)		c.2718T>G	Caucasian (1:0), AA (5:5), other (0:1)
	c.500C>T	AA (1:1), other (1:0)		c.2419G>A	Caucasian (1:0)		c.1166C>A	AA (5:5)
	c.631G>C	AA (4:0)	GYS2	c.1245C>G	AA (5:2), Hispanic (0:2)		c.988C>T	AA (5:5)
	c.775C>T	AA (3:1)		c.577G>A	Caucasian (0:1), AA (2:1), other (1:0)		c.1051G>A	AA (2:0)
	c.529C>T	Hispanic (0:1)	IL6	c.94C>T	AA (1:0), Hispanic (1:0)	PRKAA2	c.1261C>T	Hispanic (1:0)
			IL10RB	c.575G>A	AA (1:0)	PRKAR1B	c.344G>A	AA (3:1)
			MC2R	c.833T>G	AA (7:2), Hispanic (0:1)	PRKAR2A	c.721A>C	AA (1:0)

BMI percentile did not differ among racial/ethnic groups, nor did obesity, total body fat, waist circumference, or HOMA. In line with previous reports, Hispanic youth were disproportionately affected by NAFLD (26), and AAs were least affected (27). In our cohort, 36% of all patients had NAFLD, yet 43.1% of Hispanic and 29.7% of AA youth had NAFLD (**Table 2**). A total of 20.9% of patients had impaired glucose tolerance and 2.2% of patients had T2D that were similarly distributed among racial groups. Median DI was highest in the AA group [7.2 (IQR 4.2–11.3)] relative to the medians of the other racial groups (*p* = 0.043) or the entire cohort [5.7 (IQR 3.7–8.7)]. Visceral fat differed substantially by race (*p* = 0.002) and was highest in Caucasian and Hispanic groups [medians 54.2 (IQR 35.6–75.8) cm^2^ and 53 (IQR 37.2–75.4) cm^2^, respectively] and lowest in AAs [40.3 (IQR 24.1–59.2) cm^2^]. Among the cohort, 50.3% had dyslipidemia and there was an uneven distribution across races (*p* = 0.006), with 50.3%, 40.4%, 61.2%, and 38.5% of Caucasians, AAs, Hispanics, and “others” affected, respectively. The median circulating triglyceride value in our cohort was 85 (IQR 61–126) mg/dL, yet was only 64 (IQR 49–95) mg/dL in the AA group (*p* < 0.001).

### *In silico* prediction analyses

Sequencing followed by *in silico* prediction analyses identified 49 potentially meaningful variants based on the previously described criteria. Of these, 29 variants occurred in at least three patients in our cohort and were thus considered high-frequency variants and subjected to focused correlation analysis based on known gene function (**Table 3**).

### Association analyses of high-frequency variants

We performed focused association analyses among high-frequency variants within racial/ethnic groups. This entailed assessing potential effects only among clinical parameters relevant to known gene functions. Analyses included variants identified in the following genes: ATP binding cassette subfamily A member 1 (*ABCA1)*, A-kinase anchoring protein 7 (*AKAP7)*, glycogen synthase 2 (*GYS2)*, melanocortin 2 receptor (*MC2R*), phosphodiesterase 4A *(PDE4A*), phosphodiesterase 11A *(PDE11A*), peroxisome proliferator-activated receptor gamma (PPARG) and coactivator 1 beta *(PPARGC1B)*, and protein kinase cAMP-dependent type I regulatory subunit beta *(PRKAR1B*). While several other variants were identified, analyses were not performed when there were less than three individuals with any one variant within the same ethnic group. Four variants in the *ABCA1* gene were identified exclusively among AA and Hispanic patients: c.2602G>A, c.1765G>A, c.2089G>A, and c.634T>A. Presence of the c.2602G>A variant in four AA patients was associated with higher WBISI, indicating improved insulin sensitivity [5.34 ± 2.52 vs. 1.96 ± 1.39 in the AA without; *p* < 0.001]. The *ABCA1* c.2089G>A variant, present in 4 AA patients, was associated with increased visceral adipose tissue (AT) [66.0 ± 24.0 cm^2^ vs. 42.6 ± 25.3 cm^2^ in those without the variant; *p* = 0.014]. The two Hispanic patients confirmed to have the c.2089G>A variant had a mean BMI percentile of 99.67 compared to 93.58 in the entire Hispanic cohort, but the statistical analysis was underpowered for meaningful comparisons. No associations were found with the *ABCA1* c.1765G>A variant that was identified in three Hispanic patients. The *ABCA1* c.634T>A variant was confirmed in five AA patients, three of whom also harbored the *PRKAR1B* c.344G>A variant that was found to alter PKA Cα interaction with PKA regulatory subunits and was associated with a favorable lipoprotein profile (25). After adjusting for the presence of *PRKAR1B* c.344G>A, *ABCA1* c.634T>A was associated with increased total small LDL particles (556.7 ± 601.5 mg/dL vs. 536.4 ± 263.9 mg/dL without; *p* = 0.014); its associations with several other lipid biomarkers approached significance but were not supported.

The *AKAP7* c.439G>T variant was identified exclusively in AA (*n* = 9) and Hispanic patients (*n* = 5). Among AA patients, c.439G>T was associated with higher basal insulin levels [medians 60.7 (IQR 46.9–74.5) pmol/L vs. 37.0 (IQR 32.4–41.7) pmol/L without the variant; *p* = 0.001], higher 30-min insulin during OGTT [411.2 (IQR 281.7–540.7) mg/dL vs. 270.0 (IQR 226.0–313.9) mg/dL without; *p* = 0.033], and higher insulin AUC during OGTT [321.2 (IQR 222.4–420.0) mg/dL vs. 216.4 (IQR 182.8–250.0) mg/dL without; *p* = 0.038]. Hispanic youth who harbored the *AKAP7* c.439G>T variant had a median body fat percentage of 33.8% (IQR 28.2–39.4%) compared to 40.0% (IQR 38.8–41.1%; *p* = 0.035) in those without. *AKAP7* c.76G>A was confirmed in nine AA and one Hispanic patient. Among the AA cohort, it was associated with higher HDL [medians 46.4 (IQR 39.6–53.1) mg/dL vs. 38.1 (IQR 34.9–41.3) mg/dL in those without; *p* = 0.021] and lower IDL [medians 10.8 mg/dL (IQR −12.0-33.5) mg/dL vs. 35.7 (IQR 23.4–48.0) mg/dL; *p* = 0.044].

*PDE11A* c.152C>T was confirmed in 11 patients, 8 of whom were Caucasian, 1 was AA, and another 2 were Hispanic. Among Caucasian youth, the c.152C>T variant was associated with higher glucose AUC values [medians 155.2 (IQR 133.0–177.4) mg/dL vs. 131.4 (IQR 126.1–136.6) mg/dL in those without; *p* = 0.040] obtained during OGTT. HFF in the two Hispanic patients with this variant tended to be higher, but analysis was underpowered [20.0% (IQR 9.6–30.4%) vs. 9.9% (IQR 8.6–11.1%); *p* = 0.058].

Several variants in the *PPARGC1B* gene were confirmed in AA youth: c.988C>T (*n* = 10), c.1051G>A (*n* = 2), c.1166C>T (*n* = 10), and c.2718T>G (*n* = 10). Additionally, *PPARGC1B* c.2557C>T was confirmed in four Caucasian youth; one Caucasian and one “other” had the c.2718T>G variant. The *PPARGC1B* c.988C>T and c.1166C>A variants were concomitantly harbored by the same 10 patients, with one of these individuals being homozygous for both variants. Presence of *PPARGC1B* c.988C>T and c.1166C>A variants was positively associated with higher insulin AUC [medians 308.4 (IQR 221.7–395.1) pmol/L vs. 214.0 (IQR 180.0–248.0) pmol/L in those without; *p* = 0.037] during OGTT. Presence of c.2718T>G among AA was associated with older age at the time of enrollment in the clinical protocol [medians 16.5 (IQR 14.6–18.5) years vs. 13.3 (IQR 12.5–14.1) years old in those without; *p* = 0.002] and with increased subcutaneous AT [medians 678.3 (IQR 553.8–802.8) cm^2^ vs. 467.8 (IQR 417.5–518.0) cm^2^ in those without; *p* = 0.001] and increased visceral AT [median 64.6 (IQR 48.8–80.3) cm^2^ vs. 44.1 (IQR 37.7–50.5) cm^2^ without; *p* = 0.011]. The patient homozygous for c.2718T>G had 1,134.2 cm^2^ and 93.4 cm^2^ of subcutaneous and visceral AT, respectively.

*MC2R* c.833T>G was confirmed in a total of 10 patients (9 AA, 1 Hispanic), 1 of whom was homozygous for the variant. Analyses within the AA cohort revealed a tendency to be younger at the time of entry into the clinical protocol [median 11.7 (IQR 9.6–13.8) years old vs. 13.7 (IQR 13.0–14.5) years old in those without; *p* = 0.052]. The presence of the c.833T>G variant was associated with increased HFF [medians 11.3% (IQR 8.4–14.2%) vs. 8.2% (IQR 7.1–9.2%) in those without; *p* = 0.032] and SGOT [median 25.6 (IQR 21.5–29.7) vs. 21.4 (IQR 19.8–23.0) without; *p* = 0.044]. The variant was also associated with higher total cholesterol [medians 172.1 (IQR 154.9–189.2) mg/dL vs. 153.6 (IQR 147.1–160.2) mg/dL without; *p* = 0.037] and small VLDL [medians 36.4 (IQR 25.8–47.0) mg/dL vs. 24.8 (IQR 20.6–29.1) mg/dL without; *p* = 0.027]. Associations with elevated ApoB and alkaline phosphatase levels were not supported by the data (*p* = 0.089 and *p* = 0.086, respectively). The *GYS2* c.1245C>G was confirmed in seven AA and two Hispanic patients and *PDE4A* c. 163C>G was confirmed in 7 AA patients, but neither was associated with any of the clinical parameters examined.

## Discussion

Among the panel of 53 PKA signaling pathway-related genes for which we performed targeted exome sequencing, we confirmed 49 variants in our pediatric cohort that included 29 that met criteria for high-frequency allele classification (**Table 3**). In most cases, and while relatively small in sample sizes, the variants segregated neatly by racial/ethnic background, enabling us to perform association analyses that exploited the unique metabolic characteristics of each ethnic group. We did not examine non-coding variants that might also contribute to genetic regulation of the target or neighboring genes. Analyses based on sex within each racial/ethnic background were not performed due to the diminished power of any conducted analyses on these further reduced sample sizes.

It is important to note that biological sex is a major factor influencing metabolism. Additionally, sexual dimorphism in the regulation of PKA signaling is well documented in Cushing syndrome, in which prevalence in female patients is nearly twofold that in male patients (28), and in glucocorticoid regulation of metabolism (29). Numerous human and animal studies have shown that large portions of genes are differentially expressed in female and male patients in a variety of tissues including adipose, liver, and brain, with female patients tending to have more favorable metabolic profiles (30), yet in the US, more adult women than men are obese or severely obese (31). Also relevant to this study, mouse models have demonstrated extensive sex differences in PKA signaling regulation of energy balance. Global knockout of the PKA regulatory subunits RIIβ (*Prkar2b*) (32), RIIα (*Prkar2a*) (33, 34), and the catalytic subunit beta (*Prkacb*) (35) in mice all caused distinct forms of diet-induced obesity resistance that conveyed a range of sex-dependent phenotypic characteristics.

In mice, targeted deletion of *Abca1* with an adiponectin-Cre driver led to decreased adipocyte size and weight gain after high-fat diet feeding, supporting a role for *ABCA1* in adipocyte biology and in maintenance of energy balance (36). In a cohort of Northwest Mexican adults, single-nucleotide polymorphisms (SNPs) in the *ABCA1* gene as well as FTO alpha-ketoglutarate-dependent dioxygenase (*FTO)*, adrenoceptor beta 3 (*ADRB3)*, and *PPARG* were associated with increased risk of obesity (37). While *ABCA1* variant c.2602G>A was associated with improved insulin sensitivity in AAs in our cohort, the c.2089G>A variant was associated with increased visceral AT, and c.634T>A, identified only in a subgroup of Hispanic youth, was associated with elevated small LDL cholesterol levels, exemplifying the messy interplay of genetic variation.

A-kinase anchoring proteins (AKAPs) and phosphodiesterases (PDEs) represent two important classes of molecules in the regulation of PKA signaling that play roles in the subcellular localization of the PKA enzyme and regulation of cAMP levels, respectively. AKAP genes have been studied in the pathophysiology of cardiovascular disease (38), and while the role of AKAPs in metabolic syndrome and obesity is not entirely clear, their importance in the spatiotemporal regulation of PKA signaling centrally and peripherally implicates these proteins indirectly (39). Deletion of *Akap1* in mice, for example, conferred resistance to diet-induced obesity and increased energy expenditure and brown AT thermogenesis in obese mice (39). Additionally, disruption of either of the PKA type II regulatory subunits, *Prkar2b* (40) and *Prkar2a* (33), known for their interactions with AKAPs, cause resistance to diet-induced obesity in mice (by different mechanisms).

*PDE11A* defects have been linked to several cancers (adrenal, testicular, and prostate) and to major depression, bipolar disorder, and asthma, implicating specific *PDE11A* inhibitors as potential drug targets (41). Inactivating mutations in *PDE11A* cause isolated macronodular adrenal hyperplasia and Cushing syndrome that is characterized by central obesity and a panel of metabolic sequelae (42), confirming a role for this gene in metabolic regulation and energy balance. Inverse correlations between BMI and PDE activities have been observed in subcutaneous and omental AT (43) and inhibition of *PDE4* has been identified as a therapeutic target for obese women with polycystic ovary syndrome (44). In our cohort, the novel c.152C>T variant in the *PDE11A* gene was associated with higher glucose AUC in Caucasian youth.

Over 150 different variants in the melanocortin-4 receptor *(MC4R*) gene that lead to impaired receptor function have been identified, which constitute the most common cause of monogenic obesity (45). Activation of *MC4R* potently inhibits feeding without altering activity or locomotion (46). We only identified one patient with an *MC4R* variant that did not permit correlation analysis, but not surprisingly, this patient was only 8.5 years old and had a BMI percentile of 99.3. While the homozygous or multiple heterozygous variants in melanocortin-2 (*MC2R*) have been associated with familial glucocorticoid deficiency due to unresponsiveness to adrenocorticotropic hormone (ACTH), (https://omim.org/entry/202200), we found that the c.833T>G variant was associated with the younger age of patients, suggesting more severe phenotype and earlier diagnosis. The AA patients harboring the *MC2R* c.833T>G variant additionally had higher HFF and less favorable lipoprotein profiles.

No functional data were available for the *PPARGC1B* variants (ClinVar), but variants in this gene have been associated with obesity in both pediatric and adult populations. In fact, recent research into microRNA-based therapeutics inhibiting a constellation of genes, including *Ppargc1b* in mice, has shown beneficial results protecting against diet-induced obesity (47). Our data associated the c.2718T>G variant and the combination of c.988C>T and c.1166C>A variants with increased subcutaneous and visceral fat mass and elevated insulin levels, respectively. While several other variants were identified, we were not able to perform association analyses on all due to the limited number of affected individuals either in total or within ethnic groupings (**Table 3**).

A refined understanding of the genetic variations within different racial/ethnic backgrounds that predispose or protect children and teens from obesity and other metabolic complications can improve early interventions and the identification of higher-risk subgroups. Because of the multifactorial nature of obesity and metabolic diseases, screening high-risk individuals represents an additional form of intervention along with promoting healthy lifestyle choices and pharmacologic therapies. Limitations include sample sizes that in some cases did not permit definitive comparisons and the lack of evaluation of these variants in cell or animal models. Despite these shortcomings, here we identified almost 50 variants linked to PKA pathway signaling regulation of metabolism, which is a primary regulator of energy balance, adipocyte function, glucose metabolism, and lipid storage and trafficking.

## Data availability statement

All data generated and analyzed during this study are included in this published article or in the following data repository: doi: 10.6084/m9.figshare.14120888.v1 / https://figshare.com/articles/figure/PKA_NAFLD_Supplemental_Data_docx/14120888/1.

## Ethics statement

The studies involving humans were approved by the Human Investigations Committee of the Yale School of Medicine. The studies were conducted in accordance with the local legislation and institutional requirements. Written informed consent for participation in this study was provided by the participants’ legal guardians/next of kin. Written informed consent was obtained from the individual(s), and minor(s)’ legal guardian/next of kin, for the publication of any potentially identifiable images or data included in this article.

## Author contributions

MB: Investigation, Writing – original draft, Writing – review & editing, Formal Analysis. NSi: Formal Analysis, Investigation, Writing – review & editing, Methodology, Validation. FF: Investigation, Methodology, Validation, Writing – review & editing. JI: Investigation, Methodology, Writing – review & editing, Formal Analysis. SLC: Investigation, Methodology, Writing – review & editing, Project administration, Validation. SC: Writing – review & editing, Conceptualization, Funding acquisition, Supervision. NSa: Conceptualization, Funding acquisition, Writing – review & editing, Investigation, Methodology, Project administration. CS: Conceptualization, Funding acquisition, Writing – review & editing. EL: Conceptualization, Writing – review & editing, Data curation, Investigation, Project administration, Supervision, Validation, Writing – original draft.
